# Are the Organellar Genomes Useful for Fine Scale Population Structure Analysis of Endangered Plants?—A Case Study of *Pulsatilla patens* (L.) Mill

**DOI:** 10.3390/genes14010067

**Published:** 2022-12-25

**Authors:** Kamil Szandar, Sawicki Jakub, Łukasz Paukszto, Katarzyna Krawczyk, Monika Szczecińska

**Affiliations:** 1 Department of Botany and Nature Protection, University of Warmia and Mazury in Olsztyn, Plac Łódzki 1, 10-727 Olsztyn, Poland; 2 Department of Ecology and Environmental Protection, University of Warmia and Mazury in Olsztyn, Plac Łódzki 3, 10-727 Olsztyn, Poland

**Keywords:** *Pulsatilla patens*, mitogenome, plastome, genetic structure, haplotype diversity

## Abstract

*Pulsatilla patens* is a rare and endangered species in Europe and its population resources have significantly decreased over the past decades. Previous genetic studies of this species made it possible to estimate the genetic diversity of the European population and to describe the structure of chloroplast and mitochondrial genomes. The main aim of these studies was to characterize the variability of chloroplast and mitochondrial genomes in more detail at the intra-population and inter-population levels. Our study presents new organelle genome reference sequences that allow the design of novel markers that can be the starting point for testing hypotheses, past and modern biogeography of rare and endangered species *P. patens,* and adaptive responses of this species to changing environments. The study included sixteen individuals from five populations located in Northeastern Poland. Comparative analysis of 16 *P. patens* plastomes from 5 populations enabled us to identify 160 point mutations, including 64 substitutions and 96 InDels. The most numerous detected SNPs and Indels (75%) were accumulated in three intergenic spacers: *ndh*D—*ccs*A, *rps*4—*rps*16, and *trn*L(UAG)—*ndh*F. The mitogenome dataset, which was more than twice as large as the plastome (331 kbp vs. 151 kbp), revealed eight times fewer SNPs (8 vs. 64) and six times fewer InDels (16 vs. 96). Both chloroplast and mitochondrial genome identified the same number of haplotypes—11 out of 16 individuals, but both organellar genomes slightly differ in haplotype clustering. Despite the much lower variation, mitogenomic data provide additional resolution in the haplotype detection of *P. patens*, enabling molecular identification of individuals, which were unrecognizable based on the plastome dataset.

## 1. Introduction

*Pulsatilla patens* (L.) Mill, a rare and endangered species of European flora, has long been a subject of scientific research. These many years of research about the biology and ecology of this species allowed, among others: determining habitat preferences, describing the biology of this species, and developing maps with the current distribution [1,2,3,4,5]. On the other hand, the genetic study allowed for the estimation of genetic variability and identification of the population with the highest conservation values [6].

*P. patens* is an early flowering hemicryptophyte, preferring dry, well-sunny habitats and in European parts of its range, it grows most often in pine forests and thermophilic grasslands. However, over the past decades, the size of *P. patens* populations has been dramatically reduced and the present geographic range is highly fragmented [1,2,3,4,5]. In most confirmed localities, the population size is limited to several sterile specimens. The main reasons for the disappearance of this species are probably changes in land use, especially in forestry practices where efficient wildfire prevention and termination of cattle grazing in forests have led to the formation of a continuous moss layer or strongly grass-dominated vegetation, which severely hinders the regeneration of this species [4,5].

The weaker renewal of natural populations of this species may also have a genetic background which was confirmed by genetic studies of the central European population of this species. The results of this study indicate that the populations of *P. patens* are characterized by low levels of genetic diversity (*H_o_* = 0.005) and very high levels of inbreeding (*F_IS_* = 0.90) [6]. These results suggest that genetic processes occurring in populations, such as genetic drift and inbreeding may threaten the populations of *P. patens*, increasing the risk of their extinction. This genetic analysis also allowed us to distinguish three genetic groups: Baltic 1, Baltic 2, and southern populations, for which differences in allele frequencies were observed and specific alleles were identified [6]. These results suggest that the existing genetic structure reflects historical gene flow processes that led to the fragmentation of larger populations. The above assumption is validated by high values of inbreeding and low levels of migration between populations, which points to low levels of genetic exchange between populations. The split between the Baltic and the southern group of populations also coincides with the boundary between two biogeographic regions, i.e., boreal and steppic biomes in Europe [6], which implies a phylogeographic pattern.

Recent advances in sequencing technology enable us to describe the chloroplast and mitochondrial genomes of *P. patens* [7,8]. The *P. patens* plastome encodes 113 genes, excluding the second IR region, which corresponds to 79 protein-coding genes, 4 rRNAs, and 30 tRNAs. Seventeen of these genes have introns, and one copy of *ycf*1 is a pseudogene. The potential protein-coding genes are *mat*K, *ycf*3, and *ycf*4 in a large single copy (LSC), *ycf*1 at the small single copy (SSC)-IR border and *ycf*2 in IRs. The greatest intraspecific variability was demonstrated in two *ngh*I-*ndh*K, *ndh*D-*ccs*A intergenic regions and the *rps*16 gene intron, which can be successfully used in research aimed at restoring the evolutionary history of the species [7].

The mitogenome of *P. patens* revealed a multi-chromosomal structure, as the presence of three chromosomes was found with a total length of 986,613 bp [8]. The molecules are shaped by the presence of extremely long, exceeding 87 kbp, repeats and multiple chloroplast-derived regions including nearly complete inverted repeat. The mitogenome contains almost a complete set of genes known from other vascular plants with exception of *rps*10 and *sdh*3, the latter being present but pseudogenized. Analysis of long ORFs enabled the identification of genes that are rarely present in plant mitogenomes, including RNA and DNA polymerases, albeit their presence even at the genus level is variable [8].

Chloroplast DNA is characterized by the small genome, maternal inheritance of angiosperm, small selection pressure, and rapid variation of its non-coding area [9]. Until now, chloroplast genomes have been used on a much larger scale in plant research, due to their structure, maternal inheritance, and relatively conservative structure. Plastomes were most often used in phylogenetic, molecular evolution analysis, and phylogeographic studies. In contrast, data on mitochondrial genomes have not been used in plant phylogenetic and population studies so far, with exceptions of bryophytes [10], mainly due to frequent structural rearrangements, the presence of multiple long repeats, and incorporated fragments of plastid and nuclear genomes. Mitochondrial genomes are generally maternally inherited in seed plants, though some species have been shown to present paternal (some coniferous species) or biparental inheritance [11]. The uniparental inheritance of organellar genomes, together with the slow molecular evolutionary rate, explain their success as molecular markers in phylogeographic studies [12].

The recent development of novel technologies of long-read sequencing enabled the acceleration of plant mitogenomes assemblies and revealed that circular, single-chromosome mitogenomes do not dominate in vascular plant cells [13]. In recent years, more and more studies indicate the potential usefulness of mitochondrial data, mainly in phylogenetic analysis. The whole mitogenome can show an equal or even higher level of informative characters at a species-level taxonomy than the whole plastome [14].

Plant mitochondrial genomes (mitogenomes) are well known for their large size. The mitochondrial genome of plants has many unique features that distinguish it from the mitogenomes of other organisms. Compared to the relatively homogeneous size of the mitogenome of animals and fungi [15,16], respectively, the mitogenome of land plants is larger and more variable in size even between closely related species [17] and ranges from 104 kb in the moss *Anomodon rugelii* to even 11.7 Mb in *Larix sibirica* [18,19]. The differences in the size of the plant mitogenome and its structural variability are mainly related to the accumulation of non-coding sequences, especially long repeated sequences, and the presence of plastid and nuclear sequences [20,21].

The mitochondrial and plastid genomes of *P. patens* have already been studied and described in earlier studies [7,8]. The main aim of these studies was to characterize the variability of these genomes in more detail at the intra-population and inter-population levels. These studies also aimed to find whether mitochondrial genomes, the same as chloroplast genomes, could prove to be useful tools in the analysis of populations genomics and phylogeography of plants. Molecular identification of populations and individuals is especially important for reintroduction programs, enabling proper selection of transplant genotypes for specific restitution areas, and maintaining natural genetic differentiation among populations [22].

Our study presents new organelle genome reference sequences that allow the design of novel markers that can be the starting point for testing hypotheses, past and modern biogeography of rare and endangered species *P. patens,* and adaptive responses of this species to changing environments. 

## 2. Materials and Methods

### 2.1. Plant Material and DNA Sequencing

**Figure 1:**
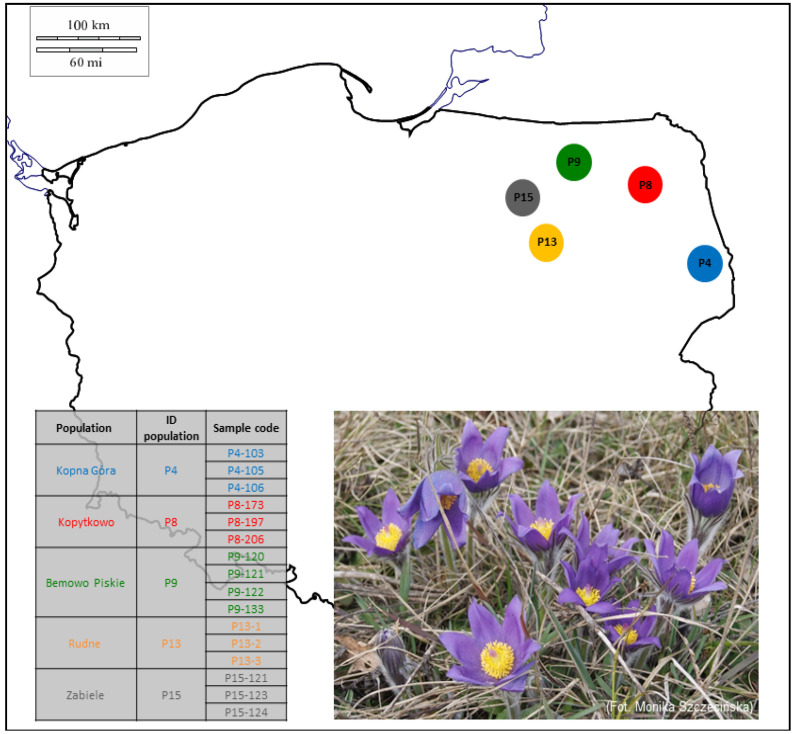
Location of sampled specimens of *P. patens*.

The study included 16 individuals from 5 populations located in Northeastern Poland (Figure 1). The samples were collected from natural habitats for the purposes of earlier studies that aimed to estimate genetic variability and genetic structure [6]. Specimens from any population are deposited in the Herbarium of the Department of Botany and Nature Protection in Olsztyn (OLS). Detailed information on the analyzed population is presented in Table 1.

### 2.2. DNA Extraction, Library Preparation, and Sequencing

Total genomic DNA was extracted from fresh tissue immediately after collection. Ca 1 cm^2^ of cleaned leaf tissue was ground with silica beads in a MiniBead-Beater homogenizer for 50 s and subsequently processed following CTAB protocol [23]. DNA quantity was estimated using the Qubit fluorometer and Qubit™ dsDNA BR Assay Kit (Invitrogen, Carlsbad, NM, USA). DNA quality was checked by electrophoresis in 0.5% agarose gel stained with Euryx Simple Safe (Eurx, Gdańsk, Poland). The genomic libraries for short-read sequencing were constructed with TruSeq DNA kit (Illumina, San Diego, CA, USA) and were sequenced using HiSeqX (Illumina) to generate 150 bp paired-end reads at Macrogen Inc. (Seoul, Korea) with 350 bp insert size between paired-ends.

### 2.3. Assembling and Mapping Organellar Genomes

The newly sequenced chloroplast genomes were assembled using NOVOplasty ver. 4.3.1 [24] with previously published *P. patens* plastome (KR297058.1) as seed and reference [7]. Geseq web server was used for annotation and minor corrections were completed using Geneious Prime 2021 software (Biomatters, Auckland, New Zealand). The single copy regions of *P. patens* mitogenome [8] were used as a reference for mapping mitogenomics reads using medium-fast algorithms as implemented in native Geneious mapper.

### 2.4. Phylogenetic Reconstruction and Variation Analyses

Haplotype networks for both datasets were calculated using a randomized minimum spanning tree method [25] based on distance obtained via *dist.DNA* function with *indelblock* model form ape 5.6 R package [26]. Phylogenetic analysis was carried out using the neighborhood-joining method (NJ) as implemented in ape 5.6 [26] with 1000 bootstrap replicates and single individual (P22-6) belonging to the “southern” genetic group [6] set as an outgroup. The optimal models for the plastome and mitogenome datasets were Hasegawa, Kishino, and Yano (HKY) and Jukes-Cantor (JC), respectively, as identified by model Test function in phangorn 2.5.3 package [27]. Mitochondrion and plastome trees were compared using cophylo function of phytools 1.0-3 R package [28].

### 2.5. Variation Detection

Assembled organellar genomes were aligned separately in two sets using MAFFT software [29]. The second copy of the plastome inverted repeat (IR) region was trimmed from the alignment. Both alignments were corrected manually and next variation calling procedure was applied using Find Variations/SNPs option implemented in Geneious Prime 2019 software (Biomatters, Auckland, New Zealand). The SNPs and InDels were detected according to the following parameters: minimum variant frequency > 0.1 and *p*-value < 10 × 10^−6^. The localization of detected variations was characterized using gff annotations and R custom script. To visualize frequency variations between populations, type of changes (SNP or InDel), and localization according to gene structure, the Circos 0.69 [30] was used.

## 3. Results

### 3.1. Plastome Variation at Species Level

The length of the complete chloroplast genomes in the 16 analyzed individuals of *P. patens* ranged from 161,836 kb (P9-122 individuals) to 161,930 (P4-103 and P4-105 individuals). The chloroplast genome of *P. patens* has a typical four-part structure: large single copy region (LSC), small single copy region (SSC), and two inverted repeat regions (IRs). Inverted repeats (IR) occurred between *rps*8 and *ycf*1. The *P. patens* plastome encodes 113 genes, excluding the second IR region, which corresponds to 79 protein-coding genes, 4 rRNAs, and 30 tRNAs. Seventeen of these genes have introns, and one copy of *ycf*1 is a pseudogene. The potential protein-coding genes are *mat*K, *ycf*3, and *ycf*4 in a large single copy (LSC), *ycf*1 in a small single copy (SSC)-IR border, and *ycf*2 in IRs. Detailed information on the structure and gene content is presented in the previous study [6].

**Figure 2:**
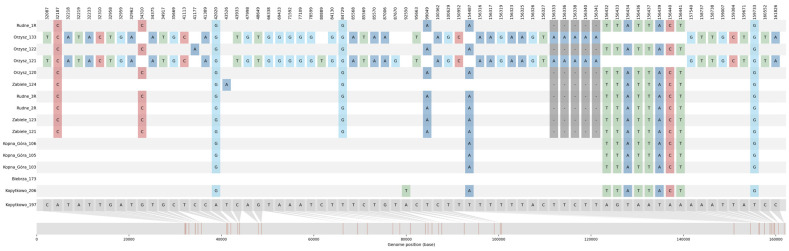
Concatenated alignment showing the 64 SNPs, with their position in the whole chloroplast genome alignment of all sequenced *P. patens* individuals.

The comparative analysis of 16 *P. patens* plastomes revealed the presence of 160 mutations, including 64 substitutions (SNPs) and 96 InDels (Figure 2, Supplementary Table S1). More than two-thirds of the SNPs (44, 68.75%) are transversions (9.38% A↔C, 37.5% A↔T, 3.12% G↔C, 18.75% G↔C) while 31.25% of the SNPs are transitions (14.06% A↔G, 17.19% C↔T). The highest numbers of SNP and InDels (93.12%) were found in the intergenic spacers and introns, only 6.88% of them were observed in the protein sequence. Nonsynonymous mutations were found in 4 genes: *mat*K, *rps*14, *atp*A, *ndh*F, (Supplementary Table S2), the others were synonymous. All the above-mentioned genes are located in single-copy regions, almost all in the LSC and only *ndh*F in the SSC region.

An intergenic spacer *ndh*D*-ccs*A with a size of 500 bp was the most variable region (20.63%) with 15 SNPs and 18 InDels (Supplementary Table S3). The second most variable region (7.5%), found between *rps*4 and *rps*16 genes, had six SNPs and six InDels. Highly similar variations (6.88%) were found in long (2079) *trn*L(UAG)—*ndh*F intergenic spacer with six SNPs and five InDels. The remaining intergenic spacers were characterized by single polymorphic sites whose prevalence did not exceed 1% in most cases.

The introns in *P. patens* plastomes were generally less variable than intergenic spacers. In non-coding regions, the most variable intron was the intron of the *rps*16 gene with three SNPs and two InDels (0.44% of variable sites). The second most variable intron (0.20%) was intron *ndh*A with one SNP and three InDels, which was 842bp longer than the intron of the *rps*16 gene. Similar variations were found in introns of the *clp*P (4 InDels).

As expected, the coding regions of the plastid genome revealed lower polymorphism than the non-coding regions. Mutations were found in 10 of 76 protein-coding genes. The most variable gene was *ndh*F with one nonsynonymous and one nonsynonymous SNP. The remaining genes had only one polymorphic site. (Supplementary Table S2).

### 3.2. Plastome Variation at the Population Level

**Figure 3:**
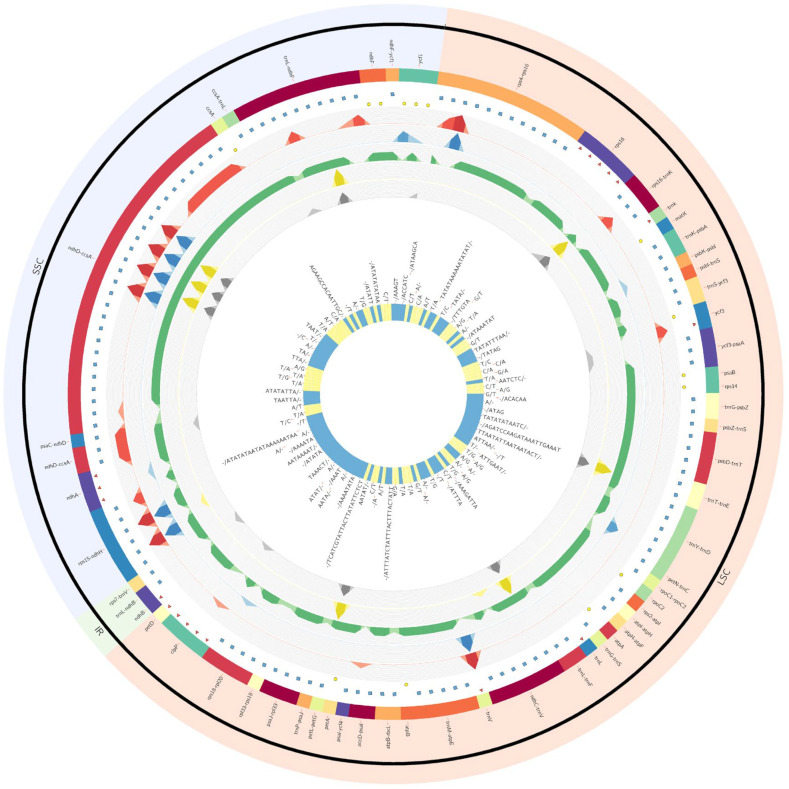
Circos plot represents mutations (SNPs and InDels) in the plastid genome of five populations of *Pulsatilla patens*. Color boxes from the outermost to the innermost indicate: (i) location name (region); (ii) location type: red triangle—intron, yellow circle—exon, blue square—intergenic region; (iii) mutations frequency of *P. patens* populations: red—Kopytkowo (P8), blue—Kopna Góra (P4), green—Bemowo Piskie (P9), yellow—Rudne (P13), gray—Zabiele (P15); (iv) mutations details; (v) type of mutation: yellow (SNPs), blue (InDels).

At the population level, the largest number of mutations in the plastome genome was found in the Bemowo Piskie population (P9) (Figure 3, Supplementary Table S1). For individuals from this population, both the greatest differences in the length of the chloroplast genome and the largest numbers of SNP (58) and InDels (74) were found. The most numerous detected SNPs and Indels (75%) were accumulated in three intergenic spacers: *ndh*D—*ccs*A (15 SNPs and 18 InDels), *rps*4—*rps*16 (5 SNPs, 5 Indels), and *trn*L(UAG)—*ndh*F (6 SNPs, 2 InDels). The remaining four populations were less variable, with a total of 48 identified mutations, including 18 substitutions and 30 InDels. The intrapopulation variation of population P8 was limited to 12 SNPs and 12 InDels, while in the P15 population 4 SNPs and 12 InDels were found. The smallest numbers of SNPs and InDels were identified for individuals of the P4 population (2 SNPs, 12 InDels) and the P13 population (2 SNPs and 8 InDels). The total number of changes per bp was greater in the plastome than in the mitogenome and it was 9.86 × 10^−4^ and 7.23 × 10^−5^, respectively.

**Figure 4:**
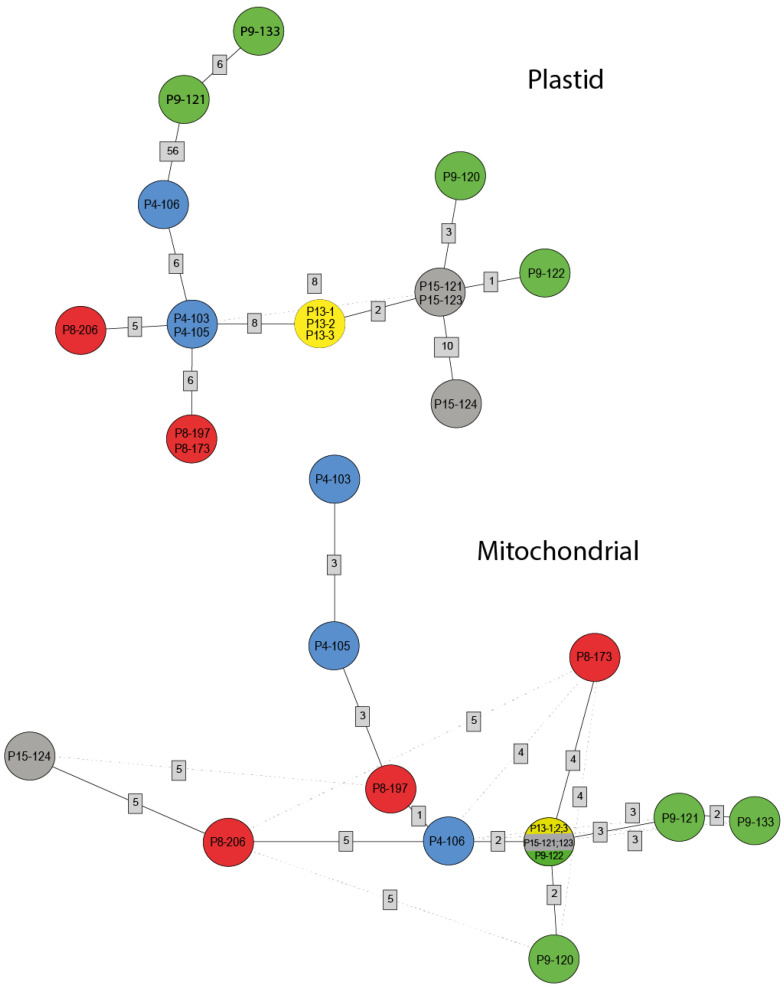
Statistical parsimony haplotype networks for chloroplast mutations (SNPs and InDels) and mitochondrial mutations (SNPs and InDels). Haplotypes signed as a sample code are shown as circles colored according to the population. Numbers in gray rectangles indicate mutations (SNPs and InDels) between nodes. Dashed lines indicate alternative pathways in the genealogy.

The haplotype network analysis distinguished 11 group cpDNA haplotypes which are divided into two groups with 1 intermediate haplotype (found in all individuals from the Rudne (P13) population) (Figure 4). The first group (shown on the left side of the haplotype network) includes five haplotypes characteristic for individuals from three studied populations (P4, P8, and P9), and the haplotypes of this group exhibit eighty-seven unique mutations, which correspond to 53.75% of all detected mutations. In this group haplotypes P9-121, and P9-133 accumulated more than half of the observed mutations—55/8%. The four haplotypes belonging to the second group (shown on the right side of the haplotype network) are more closely related to each other in comparison to the haplotype of the first group (14.41% of all mutations). The most heterogeneous was the population Bemowo Piskie (P9), where each individual presented a different haplotype. Moreover, for individuals from this population, both the greatest differences in the length of the chloroplast genome and the largest numbers of SNP (60) and InDels (74) were found. Most of these SNPs were found in two haplotypes (P9-121, P9-133), which differed from the other two (P9-120, P9-123) by the presence of 61 SPNs and 59 InDels. The other two haplotypes (P9-120, P9-123) were more similar to haplotypes representing other populations. Each of the three analyzed populations (P8, P15, and P4) was characterized by two different haplotypes. In this group of populations, the highest number of mutations (12 SNP and 6 InDels) between individuals was detected for population P8. In population P15 we observed two haplotypes differing by the presence of four SNPs and four InDels and, respectively, in population P4 haplotypes differed by six InDels. On the other hand, in population P13 we observed only one haplotype.

### 3.3. Mitogenome Variation at Species Level

The mitochondrial genome of *P. patens* revealed a multichromosomal structure and contains three linear chromosomes of total length 986,613 bp. Detailed information on the structure and gene content is presented in the previous study [8]. Based on a previous study [8] 14 single-copy regions, not split by repeats or plastid-derived sequences (MTPT), were selected as a potential resource for population-scale studies. Most of these regions (eight) were located at the longest chMt1chromosome while the remaining six were located at chMt2 (four) and chMt3 (two) chromosomes.

The analyzed regions of the mitochondrial genome turned out to be less variable compared to the plastid genome with only 24 changes (8 SNPs and 16 InDels) (Table 2, Supplementary Table S4).

The most mutations, five SNPs and six InDels (45.83%), were found in the analyzed region located on chromosome chMt1 while the lowest number of mutations, one SNP and five InDels (25%) was found on the regions of chromosome chMt2. However, chromosome chMt3 was the most variable in terms of the number of mutations per number of bp (1.84 × 10^−4^). The largest number of mutations, 6 (26.1%), was revealed in the *nad*2 intron on chromosome chMt3 (5 InDels and 1 SNP) (Supplementary Table S5).

### 3.4. Mitogenome Variation at Population Level

**Figure 5:**
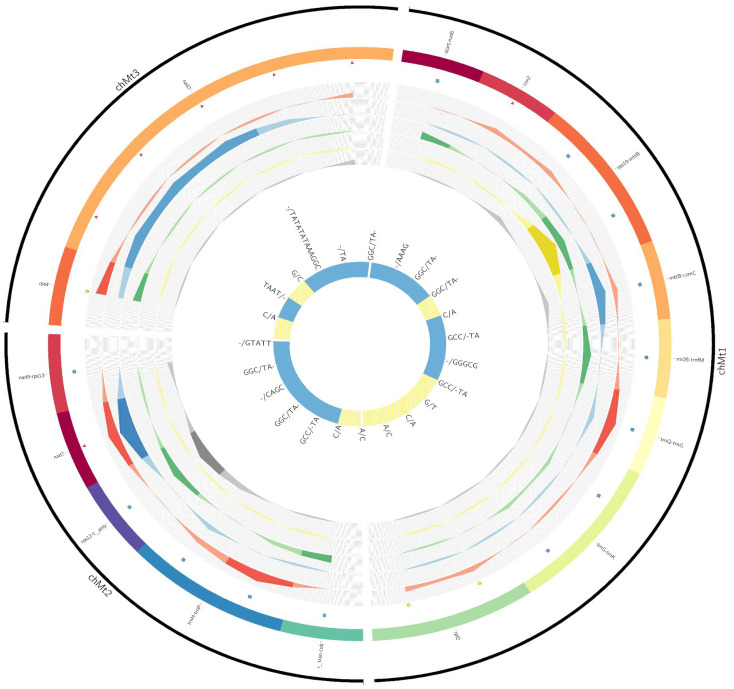
Circos plot represents mutations (SNPs and InDels) in the mitochondrial genome of five populations of *Pulsatilla patens*. Color boxes from the outermost to the innermost indicate: (i) location name (region); (ii) location type: red triangle—intron, yellow circle—exon, blue square—intergenic region; (iii) mutations frequency of *P. patens* populations: red—Kopytkowo (P8), blue—Kopna Góra (P4), green—Bemowo Piskie (P9), yellow—Rudne (P13), gray—Zabiele (P15); (iv) mutations details; (v) type of mutation: yellow (SNPs), blue (InDels).

At the population level, the largest number of mutations in the mitogenome was found in the Kopytkowo population (P8) (eight InDels, three SNPs) as seen in Figure 5. A very similar number of mutations was observed in the Kopna Góra population (P4) (eight InDels, two SNPs) and Bemowo Piskie population (P9) (five InDels, four SNPs). However, the Rudne population was characterized by only one mutation. (Supplementary Table S5). Analyses distinguished 11 haplotypes, and intra-specific mitochondrial DNA divergence in *P. patens* is presented in Figure 4. The analysis did not divide sixteen individuals from five populations into subgroups corresponding to their geographical location. These results do not correspond to the data for chloroplast DNA. The center consists of all three haplotypes from the P13 population, two from the P15 population (P15-121, P15-123), and one from the P9 population (P9-122) grouping together. The remaining haplotypes do not group together and are located at relatively short distances around the center of the haplotype network. As in the cpDNA results, the mitochondrial genome distinguished the same number of haplotypes in the P9, P13, and P15 populations. The P9 population again turned out to be the most heterogeneous with four different haplotypes that formed exactly the same two groups as for cpDNA results. However, the difference was observed for the P8 and P4 populations, wherein each of the three different haplotypes was observed, while the cpDNA analysis distinguished only two haplotypes.

### 3.5. Phylogenetic Analysis of Mitochondrial and Chloroplast Datasets

**Figure 6:**
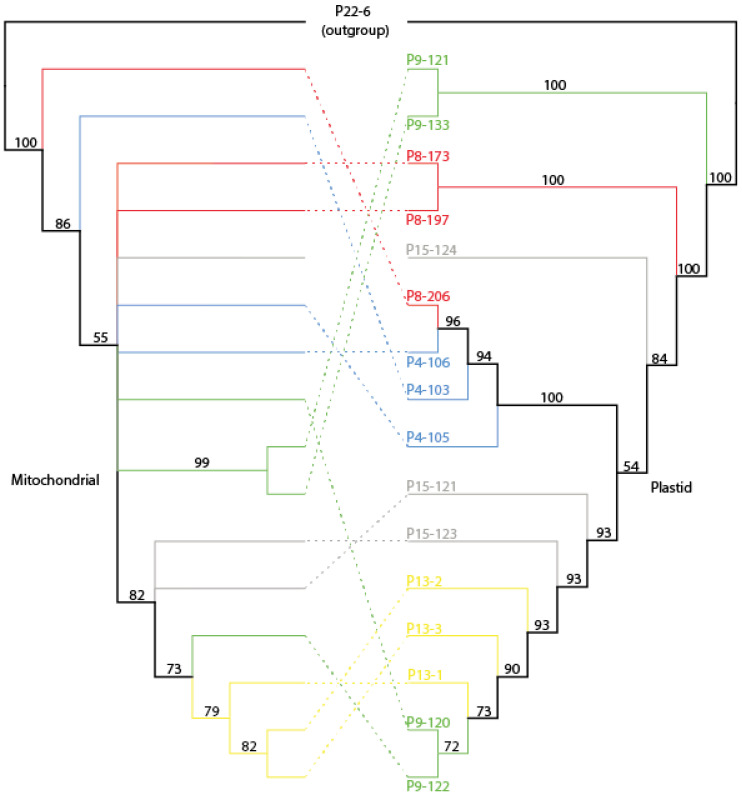
Comparison of NJ trees based on mitochondrial and plastid datasets. The bootstrap values are given above supported branches.

The chloroplast dataset resolved trees more efficiently than mitogenome data, with only one clade with a low bootstrap value (Figure 6). The comparison of phylogenetic trees based on mitochondrial and plastid datasets showed differences in topologies. In both phylogenetic trees, individual P22-6 from the Ukrainian population, belonging to the “Southern” genetic group [6] was used as a root and all individuals of “Baltic” populations formed common, well-supported clades (100% BS support). In the mitochondrial tree, individual P8-206 from the Kopytkowo population was the most similar to the outgroup, followed by a P4-103 from the Kopna Góra population. The most internally homogeneous and at the same time the most distant was the P-13 population (Rudne), in which individuals formed a common, well-supported clade (79% BS support). With the exception of individuals from the P-13 population, none of the others grouped together according to their area of distribution. In the phylogenetic tree based on the chloroplast dataset, the closest to the outgroup were two individuals from the Bemowo Piskie population (P9-121, P9-133), which were grouped together. Interestingly, the remaining individuals from this population (P9-120, P9-122) also grouped together but were the most distant in the phylogenetic tree. As in the phylogenetic tree made from the mitochondrial dataset, individuals from the P-13 population grouped together, but were joined by individuals from the P9 population (P9-120, P9-122). A similar situation takes place in the P4 population, the individuals of which are grouped together and joined by the individual P8-206 from the Kopytkowo population. The remaining individuals did not cluster together according to their area of occurrence.

## 4. Discussion

Until now, chloroplast markers and complete chloroplast genomes of plants have been widely used and have made significant contributions to phylogeny reconstruction at different taxonomic levels in plants [31,32] including species of the genus *Pulsatilla* [33,34,35,36]. Plastid phylogenomic analyses, among others, showed that the species of *Pulsatilla* formed a monophyletic group, and should not be synonymized with the genus *Anemone*. However, the analysis of the variability of chloroplast genomes at the inter- and intrapopulation levels has not been widely studied so far. Generally, in lower taxonomic units there is probably less variation at the level of the plastid genome, which is mainly observed in hotspot regions. 

*P. patens* is a critically endangered species in Europe and its population resources have been dramatically reduced in recent decades. Obtaining genomic information about the infraspecific variation of the plastid and the mitochondrial genome is the next step of the genetic study of this species. In a previous study, we estimated the genetic diversity of the European population and sequenced and assembled the mitogenome and plastome of this species [6,7,8,37]. The size of the chloroplast genome of analyzed individuals ranged from 161,836 kb to 161,930 which falls within the range of the values obtained for other *Pulsatilla* species [7,33,34,35,36]. Newly sequenced plastomes, similar to other *Pulsatilla* species, showed high sequence similarity across coding regions (only 6.88% of them were observed in protein sequence) and more variability in non-coding regions (95.12%). Previous studies made it possible to identify nine divergent hotspots regions including intergenic spacer regions (*rps*4-*rps*16, *rps*16-*mat*K, *ndh*C-*tnr*V, *psb*E-*pet*L, *ndh*D-*ccs*A, *ccs*A-*ndh*F) in nine analyzed *Pulsatilla* species [7,36]. The same regions were revealed to be the most variable at the intraspecific level of *P. patens*. The most variable region (20.63%) was an intergenic spacer *ndh*D*-ccs*A and region between *rps*4 and *rps*16 genes (7.5%). Point mutations resulting from DNA replication errors should be evenly distributed in single-copy regions of the plastome. Many hotspots in the *P. patens* plastome are localized in the vicinity of the most variable *ycf*1 gene and its pseudogenized copy. Such uneven distribution of point mutations in the plastid genome also occurs in other plant groups. In the genus *Lathyrus,* the rate of mutations in several regions of the plastome was more than 20 times higher than in the remaining regions [38]. Fast evolving regions in species of the genera *Pelargonium*, *Plantago,* and *Silene* testify to the presence of localized hypermutations [39,40]. It is believed that localized hypermutations are induced by a higher number of error-prone double-strand break repairs [38,39].

Comparative analysis of 16 *P. patens* plastomes from 5 populations belonging to one genetic group, enabled the identification of 160 point mutations, including 64 substitutions and 96 InDels (Table S1). Since our knowledge of plastome variation at fine geographic scales is very limited, it is hard to conclude if these values are low or high, especially considering *P. patens* extinction risk status. The analysis of 3 chloroplast genomes of *Abies sachalinensis* from distant populations of Hokkaido Island revealed 94 mutations, including 52 substitutions [41], but all 3 individuals were previously clustered in different genetic groups [42]. In contrast, analyzed individuals of *P. patens* were sampled from populations belonging to the same genetic cluster [6,37]. A higher number of SNPs and InDels (162 and 92, respectively), were identified among 20 wild and cultivated *Ricinus communis* accessions, but again in this study sampling was much wider [43], but again, it is a widespread and cultivated species. The total number of analyzed accessions sometimes does not correlate with the number of detected SNPs and InDels, as in the case of the comparative analysis of *Euonymus maackii*, where 652 SNPs and 65 InDels were detected between two individuals [44]. However, until more data on intraspecific plastome variation of species similar to *P. patens* in terms of biology, ecology and evolutionary history will be available, the obtained results are hardly comparable with those previously published.

In addition to information on intraspecific plastome variation presented in this study, results show intrapopulation differentiation of *P. patens* haplotypes. The application of the complete chloroplast genome to study fine-scale genetic structure was not explored so far. Although in this analysis we used a small number of individuals from populations, the chloroplast genome enabled molecular identification in 11 out of 16 individuals. The picture of intra-population genetic variability obtained in these studies does not reflect the results of previous analyses, based on SSR markers [6]. Based on microsatellite analysis the studied populations were characterized by a very low percentage of heterozygous individuals and it was also confirmed by a high inbreeding coefficient. The high level of observed and expected heterozygosity was detected in population P15 (Ho = 0.102, He = 0.740), which in these studies did not turn out to be the most variable. Within this population, analysis of cpDNA and mtDNA regions identified two haplotypes (Figure 4), differed by the presence of four SNPs and four InDels in cpDNA, and six InDels in mtDNA. In the population P4, characterized by three distinct plastid and mitochondrial haplotypes, the SSR markers did not reveal any heterozygosity. On the other hand, no variation in organellar genomes of the moderately variable population P13 (parametry) was found. The different and partially contrasting patterns of variation revealed by nuclear microsatellites and organellar genome could be expected, as these molecules have different mechanisms of dispersal, inheritance, and DNA repairing. Incorporating uniparentally inherited markers into nuclear-based microsatellites dataset for population variation analysis of *Pulsatilla patens* could enrich polymorphism information and improve conservation strategies.

The chloroplast genome enabled molecular characterization of the analyzed populations, which confirms limited gene flow by seeds between populations. The seeds of this species usually fall near the mother plant, some may be wind dispersed. However, it seems that long-distance seed transport occurs sporadically [4]. The haplotype network analysis divided haplotypes into two groups with one intermediate haplotype (found in all individuals from the Rudne (P13) population) which did not correspond to their geographical location.

Compared to the genetic variability of the chloroplast genome, the analyzed regions of the mitochondrial genome of *P. patens* turned out to be less variable (8 SNP and 16 InDel), which is currently explained by a highly efficient repair mechanism and, therefore, a generally low mutation rate in the mitochondrial genomes of plants [45]. Similarly to other plant species also in the pasque-flower most of the observed mutations were accumulated in non-coding sequences, only SNP was observed in the *rps*4 gene. The mitogenome dataset, which was more than twice as large as the plastome (331 kbp vs. 151 kbp) revealed eight times fewer SNPs (8 vs. 64) and six times fewer InDels (16 vs. 96). Slower mutational rate of mitogenomes than plastomes seems to be common in plants. Many studies report that the substitution rate of the plastome was three times higher than that of the mitogenome in angiosperms and twice as high as the substitution rate of the mitogenome in gymnosperms [46]. However, the comparative mitogenomic data at the intraspecific level is still very limited. Only one SNP and three InDels were found between two European and North American mitogenomes of *Nowellia curvifolia*, while European accessions were identical [47]. Analysis of complex plant mitogenomes is challenging, but the latest advances in long-read sequencing enabled assembling complex, large mitogenomes [48], but studies focus mainly on high-ranked taxa.

Despite the much lower variation, mitogenomic data provide additional resolution in the haplotype detection of *P. patens*, enabling molecular identification of individuals, which were unrecognizable based on the plastome dataset. Application of both organellar genomes seems to be especially useful in fine-scale population structure analyses such as seed-based gene flow and demographic parameters.

## 5. Conclusions

The newly sequenced plastomes and single copy regions of mitogenome of endangered *Pulsatilla patens* revealed variation on both inter- and intrapopulation levels, despite that the earliest studies based on SSR and ISJ (Intron Splice Junction) markers revealed low polymorphism of analyzed samples. The chloroplast genome, despite being more variable than mitochondrial, identified the same number of haplotypes—11 out of 16 individuals, but both organellar genomes slightly differ in haplotype clustering. The application of complete organellar genomes in fine-scale population genetics has great potential, but more comparative data are needed for the proper evaluation of obtained variation values. However, our results show that the inclusion of organellar genomes in population genetics studies could provide additional information, which is not always congruent with nuclear-based markers. Further genomic-scale population studies based on RNAseq or RADseq methods should incorporate genetic information from relatively small organellar genomes, but not necessarily merge it into one dataset, as these molecules have different mechanisms of inheritance and DNA repairing.

## Figures and Tables

**Table 1:** Characteristics of five analyzed populations of *P. patens*.

Locality	ID Population *	Geographical Coordinates	Sample Code	GenBank Accession Number (Plastome)	GenBank Accession Number (Mitogenome)		
					chMt1	chMt2	chMt3
Kopna Góra	P4	54°01′ N 23°12′ E	P4-103 P4-105 P4-106	OP115677 OP115678 OP115679	ON454164 ON454165 ON454166	ON454180 ON454181 ON454182	ON454196 ON454197 ON454198
Kopytkowo	P8	53°58′ N 23°56′ E	P8-173 P8-197 P8-206	OP115680 OP115681 OP115682	ON454167 ON454168 ON454169	ON454183 ON454184 ON454185	ON454199 ON454200 ON454201
Bemowo Piskie	P9	53°45′ N 21°59′ E	P9-120 P9-121 P9-122 P9-133	OP115683 OP115684 OP115685 OP115686	ON454170 ON454171 ON454172 ON454173	ON454186 ON454187 ON454188 ON454189	ON454202 ON454203 ON454204 ON454205
Rudne	P13	53°23′ N 21°35′ E	P13-1 P13-2 P13-3	OP115687 OP115688 OP115689	ON454174 ON454175 ON454176	ON454190 ON454191 ON454192	ON454206 ON454207 ON454208
Zabiele	P15	53°27′ N 21°10′ E	P15-121 P15-123 P15-124	OP115690 OP115691 OP115692	ON454177 ON454178 ON454179	ON454193 ON454194 ON454195	ON454209 ON454210 ON454211

* Population ID is given after the article Szczecińska et al. 2016.

**Table 2:** Concatenated alignment showing the 8 SNPs on 3 chromosomes, with their position in the mitogenome alignment of all sequenced *P. patens* individuals. If an SNP is located within a gene or intron the corresponding gene name is given in the first row. Non-synonymous SNPs are marked by an asterisk (*).

Gene							*rps*4	*nad*2 (Intron)
Position Individual	ChMt1—22156	ChMt1—110241	ChMt1—110242	ChMt1—167288	ChMt1—167297	ChMt2—30884	ChMt3—4214	ChMt3—30033 *
P4-103	C	G	C	A	A	C	C	C
P4-105	C	G	C	A	A	C	C	G
P4-106	C	G	C	A	A	C	A	C
P8-173	C	G	C	A	A	C	A	A
P8-197	C	G	C	A	A	C	A	A
P8-206	C	G	C	C	C	C	C	G
P9-120	C	G	C	A	A	C	C	G
P9-121	A	G	C	A	A	A	A	G
P9-122	C	G	C	A	A	C	C	G
P9-133	A	T	A	A	A	A	A	G
P13-1	C	G	C	A	A	C	C	G
P13-2	C	G	C	A	A	C	C	G
P13-3	C	G	C	A	A	C	C	G
P15-121	C	G	C	A	A	C	C	G
P15-123	C	G	C	A	A	C	C	G
P15-124	C	G	C	A	A	C	C	G

## Data Availability

The chloroplast and mitogenome sequences are deposited in GenBank with accession numbers: chloroplast genome; OP115677-OP111569, mitogenome; ON454164-ON454211.

## Supplementary Materials

The following supporting information can be downloaded at: https://www.mdpi.com/article/10.3390/genes14010067/s1, Table S1: Number of SNP and InDels detected in chloroplast genome in analyzed population.; Table S2: Number of SNP detected in chloroplast and mitochondrial CDS.; Table S3: Mutation frequency in particular regions in chloroplast genome (gray shaded lines are introns; blue exons; orange introns and exons).; Table S4: Number of SNP and InDels detected in mitogenome per population; Table S5: Total number of SNPs and InDels detected in mitochondrial genome.
